# Inflammatory Monocytes and Neutrophils Are Licensed to Kill during Memory Responses *In Vivo*

**DOI:** 10.1371/journal.ppat.1002457

**Published:** 2011-12-29

**Authors:** Emilie Narni-Mancinelli, Saidi M'Homa Soudja, Karine Crozat, Marc Dalod, Pierre Gounon, Frédéric Geissmann, Grégoire Lauvau

**Affiliations:** 1 Institut National de la Santé et de la Recherche Médicale Unité 924, Groupe Avenir, Université de Nice-Sophia Antipolis, Valbonne, France; 2 Université de Nice-Sophia Antipolis, UFR Sciences, Nice, France; 3 Centre d'Immunologie de Marseille-Luminy, Université de la Méditerranée, Institut National de la Santé et de la Recherche Médicale Unité 631, Centre National de la Recherche Scientifique, Unité Mixte de Recherche 6102 (CNRS-UMR), Marseille, France; 4 Albert Einstein College of Medicine, Department of Microbiology and Immunology, Bronx, New York, United States of America; 5 King's College London, Centre for Cellular and Molecular Biology of Inflammation, London, England, United Kingdom; Stanford University, United States of America

## Abstract

Immunological memory is a hallmark of B and T lymphocytes that have undergone a previous encounter with a given antigen. It is assumed that memory cells mediate better protection of the host upon re-infection because of improved effector functions such as antibody production, cytotoxic activity and cytokine secretion. In contrast to cells of the adaptive immune system, innate immune cells are believed to exhibit a comparable functional effector response each time the same pathogen is encountered. Here, using mice infected by the intracellular bacterium *Listeria monocytogenes*, we show that during a recall bacterial infection, the chemokine CCL3 secreted by memory CD8^+^ T cells drives drastic modifications of the functional properties of several populations of phagocytes. We found that inflammatory ly6C^+^ monocytes and neutrophils largely mediated memory CD8^+^ T cell bacteriocidal activity by producing increased levels of reactive oxygen species (ROS), augmenting the pH of their phagosomes and inducing antimicrobial autophagy. These events allowed an extremely rapid control of bacterial growth *in vivo* and accounted for protective immunity. Therefore, our results provide evidence that cytotoxic memory CD8^+^ T cells can license distinct antimicrobial effector mechanisms of innate cells to efficiently clear pathogens.

## Author Summary

The immune system comprises white blood cells that belong to the innate or the adaptive immune arms. Adaptive immune cells such as T and B lymphocytes can give rise to memory cells which mediate long-lived immunity against pathogens. During a recall infection, innate immune phagocytic cells such as monocytes and neutrophils can be critical to kill microbial pathogens inside infected tissues. Whether and how such antimicrobial features of phagocytic cells of the innate immune system are modulated during a memory response in a vaccinated host is not known. The present report shows that cytolytic memory T lymphocytes, an important subpopulation of effector T cells, can drastically enhance the functional killing capacities of monocytes and neutrophils for optimized pathogen clearance from infected hosts. These phagocytes exhibit enhanced generation of oxidative burst, increased phagosomal pH and autophagy, three mechanisms that lead to intracellular pathogen death. This result is important since it suggests that modulating innate immune cells effector activities could be an interesting strategy to enhance vaccine efficacy.

## Introduction

Immunological memory is a central feature of the adaptive immune system and relies on the maintenance of antigen-experienced B cells and T cells. In general, a memory immune response is faster and stronger than a primary response because of the increased frequency, improved functional characteristics, and preferential localization of the memory cells in peripheral tissues [1]–[2]. Memory CD8^+^ T cells represent one major effector arm of the adaptive immune system that maintains long-lived protective immunity against intracellular bacteria, protozoa, and viruses [1]. Although CD8^+^ T cells are mainly appreciated for their capacity to kill infected cells, they can express various effector mechanisms that potentially contribute to host defense against infections [3]. Former reports have suggested that the antimicrobial potential of CD8^+^ T lymphocytes is also reflected in their ability to rapidly produce inflammatory cytokines such as IFN-γ, TNF-α and CCL3 that promote the control of the growth of intracellular pathogens such *as Francisella tularensis, Leishmania major or Listeria monocytogenes (Lm)* by phagocytes [4]–[6]. In the case of a memory response, both the increased numbers of recruited phagocytes and their faster or chronic activation allow for an optimized clearance of intracellular pathogens [6]–[9]. This view is based on the assumption that phagocytes usually exhibit a qualitatively and quantitatively comparable response each time the same pathogen is encountered. However, it is not known whether and how the antimicrobial activities of innate phagocytes that are expressed during a primary immune response are modulated during recall infections.

Phagocytes such as macrophages, neutrophils and monocytes play a critical role during primary infections to fight intracellular pathogens. Both neutrophils and macrophages are well-established as important cellular effectors of innate immune defense, and it is clear that circulating monocytes also contribute significantly to the defense against a range of microbial pathogens [10]–[11]. Phagocytic cells recognize and engulf pathogens by expressing specific receptors of microbial products (pattern recognition receptors, PRRs), of the complement system or of antibodies complexed to pathogen-derived molecules [12]. Following microbial internalization, early phagosomes undergo numerous steps of maturation that are concomitant with important changes of their associated proteins and luminal pH acidification. Acidification results from the fusion of phagosomes with pre-existing lysosomes, leading to the activation of hydrolytic enzymes which act optimally at acidic pH (4.5–5.0) to enable the degradation of micro-organisms [13]. In addition to acidification, another critical mechanism involves recruitment to the phagosomal membranes of the nicotinamide adenine dinucleotide phosphate (NADPH) oxidase complex subunits which in turn generate the reactive superoxide ions that accumulate in the phagosomal lumen [14]. Superoxide is further converted to several highly toxic radical oxygen species (ROS) that degrade phagosomal content and facilitate pathogen killing [15]–[16]. Generation of ROS also induces phagosomal pH increase which subsequently activates neutral proteases that digest and kill microorganisms. Upon activation, phagocytes generate other important antimicrobial effector molecules such as reactive nitrogen species (RNS) produced by the inducible nitric oxid synthase (iNOS) that interact with ROS to exert very toxic effects on intraphagosomal engulfed microorganisms [17]. Collectively, these antimicrobicidal effectors activities provide a toxic phagosomal environment that efficiently limits pathogen proliferation. However, some pathogens including bacteria such as *Listeria, Shigella, Rickettsia* and group A *Streptococcus* (GAS) have evolved strategies to quickly escape from this hostile phagosomal environment to the more hospitable milieu of the host cell cytosol [18]. After escape into the cytosol, some of these bacteria can be trapped in large cytosolic vacuoles and ultimately be degraded within autolysosomal compartments [19]–[21]. This process, known as autophagy, has been more recently described as an intrinsic host defense mechanism for recognition and elimination of intracellular cytosolic or vacuolar pathogens [19].

In a previous study using mice intravenously inoculated with *Lm* as an infection model, we have shown that innate phagocytes can be activated by the Macrophage Inflammatory Protein 1α (MIP-1α/CCL3) that is secreted by memory CD8^+^ T cells upon antigen-driven reactivation [6]. CCL3 induced a rapid TNF-α secretion by innate inflammatory monocytes, which further promoted the production of ROS by both monocytes and neutrophils. We also found that ROS generation depended on CCL3 and was required for killing of *Lm*. In the present work, taking advantage of the same experimental settings in which the vast majority of bacteria were found inside phagocytic cells of the spleen [22]–[23], we investigated the modulation of the antimicrobial effector activities expressed by innate phagocytes in the course of the secondary infection and the impact this have on the clearance of the bacteria from infected tissues.

## Results

### A major role for CCL3-mediated reactive oxygen species (ROS)-dependent killing of bacteria in protection of immunized mice

**Figure 1:**
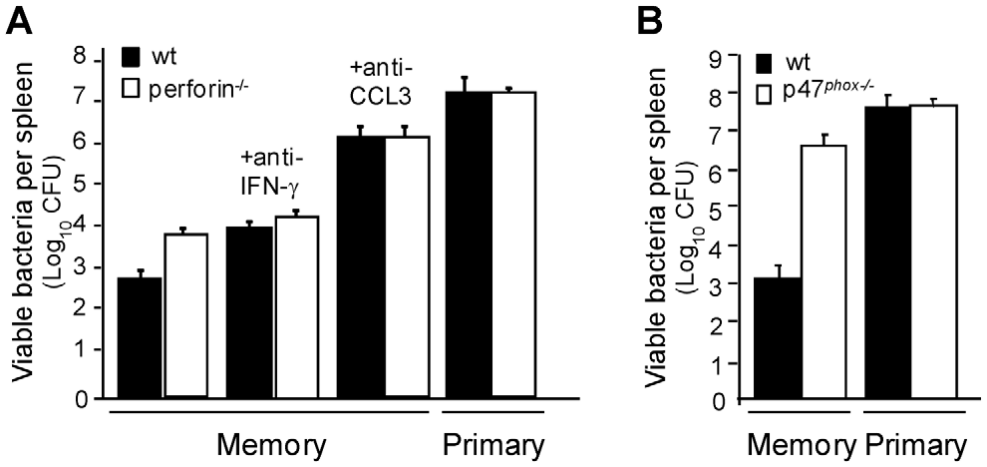
CCL3/ROS^+^-mediated killing of bacteria is a major mechanism of protection in memory mice. Wt (black bars) (**A–B**) and perforin^−/−^ mice (white bars) (A) or p47*^phox^* ^−*/*−^ mice (**B**) were injected with PBS or immunized with *Lm*. One month later mice were treated or not with anti-CCL3 serum or anti-IFN-γ mAb and subsequently challenged with 3×10^5^ wt *Lm* for 2 days. Data show the number of bacteria CFUs (mean±SE) in a pool of two independent experiments with n = 6–9 mice per group.

CD8^+^ T cells usually protect against intracellular microbes by the recognition and the lysis of infected cells, via a perforin and/or a Fas/Fas-ligand-mediated mechanism of cytolysis. We recently demonstrated in the mouse *Lm* infection model that memory CD8^+^ T cells are also able to protect by orchestrating the activation of effector cells of the innate immune system via the release of the chemokine CCL3/MIP1-α [6]. Indeed, the transfer of CCL3-deficient effector [24] or memory [6] CD8^+^ T cells to infected mice does not confer protective immunity to these mice. Moreover, the protection achieved by wt memory CD8^+^ T cells infused into CCL3^−/−^ recipient mice is totally abrogated upon CCL3 neutralization [6]. At least 2 recent reports have documented that memory CD8^+^ T cells induced in a bacterial and a viral infection indeed can produce high levels of CCL3 and that expression of this gene is enhanced in highly differentiated, long-lived memory cells that can confer immunological protection [25]–[26]. We further compared this mechanism to the classical CD8^+^ T cell-dependent effector mechanisms. For this, wt or perforin-deficient mice were immunized by infection with *Lm* (then referred to as “memory mice”), and 3 weeks later some animals were treated with anti-CCL3 serum or anti-IFN-γ neutralizing mAb before the challenge infection (Figure 1A). While animals lacking perforin exhibited only a modest increase in susceptibility (∼10 fold) compared to wt memory mice [27], none of the anti-CCL3-treated groups controlled the challenge infection. These mice had more than 10^6^ bacteria in the spleen, a number close to that found in primary and in memory p47^phox−/−^ infected mice that lack functional NADPH oxidase complexes and cannot produce ROS (Figure 1B). Interestingly, when memory mice were treated with a neutralizing Ab against IFN-γ, an important immunomodulatory cytokine of effector and memory CD8^+^ T cells, only about a 10 fold loss of protection was observed in wt mice (Figure 1A) [28]. Thus, whereas perforin- and IFN-γ accounted for a significant degree of protection in wt memory animals, the current results revealed a substantially greater role for CCL3 and ROS in protective memory responses to *Lm*.

### Improved antimicrobial vacuolar effector functions in phagocytes during memory responses

**Figure 2:**
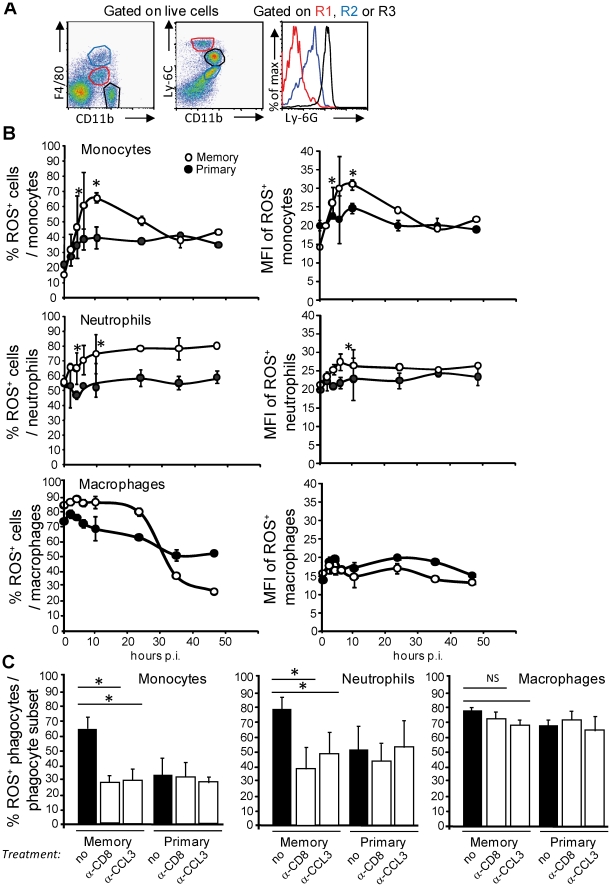
Increased frequencies of ROS^+^ inflammatory monocytes and neutrophils that generate higher levels of ROS during the secondary infection. (**A**) Spleen cells from wt mice (3/group) were independently analyzed by FACS for surface expression of F4/80, CD11b, Ly-6C and Ly-6G. Data show representative FACS profiles in a representative (out of 2) experiment. Red gate: inflammatory Ly6C^+^ monocytes, black gate: neutrophils, blue gate: macrophages. (**B**) Mice (9–10 per group) primarily injected with PBS (closed circles, primary) or 0.1xLD_50_ (3×10^3^) wt *Lm* (open circles, secondary) were challenged 30 days later with 10xLD_50_ (3×10^5^) wt *Lm*. At the indicated times after challenge, spleen cells were restimulated with Heat Killed *Lm* (HKLM) in the presence of hydroethidine and analyzed by FACS for CD11b and Ly-6C expression. Data show the number (mean +/− SE) (left panels) and the MFI (mean +/− SE) (right panels) of ROS-producing inflammatory monocytes (upper panel), neutrophils (middle panel) and macrophages (bottom panel) per total gated inflammatory monocytes, neutrophils or macrophages and are representative of a pool of 2–3 replicate experiments. (**C**) Primary and memory mice (10/group) were treated 30 days later with a control isotype or serum (black bars), anti-CD8 or anti-CCL3 (white bars) and challenged with 3×10^5^ wt *Lm*. At 10 hrs after the infection, the frequencies of ROS-producing cells were analyzed as in (**B**). Data result from the pool of 2 independent experiments. P (*<0.05) values were calculated in (B–C) with n = 9–10.

To study the modulation of the effector functions of phagocytes during a memory response, we first defined the cytofluorometry gating strategies that allowed us to identify the major innate phagocyte populations of the spleen. While neutrophils (CD11b^high^F4/80^low^Ly-6G^high^) and macrophages (CD11b^med^F4/80^high^Ly-6G^med^) are well-defined in the literature (Figure 2A), blood-monocytes that enter inflamed tissues and express antimicrobial functions have been variously described as inflammatory monocytes, [29], TNF-α/iNOS producing-dendritic cells (Tip-DCs) [30] or mononuclear phagocytic cells (MPCs) [6] depending on the experimental context. Since all of these cells exhibit a comparable cell-surface phenotype (CD11b^med/high^F4/80^med^Ly-6G^low^CX3CR1^low^CD11c^low^Ly-6C^high^), produce TNF-α, RNS and ROS (Figure 2A, S1), it is likely that inflammatory monocytes, Tip-DCs and MPCs are the same cell population, which we refer to as inflammatory and/or ly6C^+^ monocytes.

In addition to the generation of ROS, phagocytic cells also express other important antimicrobial activities within their phagosomes during activation such as the generation of reactive nitric species (RNS) and the rapid modulation of the pH of the phagosomal lumen [31]. Since the enzymes that produce NO (the inducible nitric oxide synthase, iNOS) and ROS (the NADPH oxidase) can be activated by the release of IFN-γ and TNF-α, and because levels of these cytokines are much higher during the memory response, we monitored the activation of both enzymatic complexes during a primary as compared to a memory immune response. Mice injected with PBS or with Lm were challenged a month later with *Lm* (primary and memory responses, respectively), and the frequencies and mean fluorescence intensities (MFI) of ROS-producing and iNOS-expressing cells were measured by flow cytometry (Figure 2B–C, S2 and S3). The numbers and frequencies of ROS-producing inflammatory monocytes and neutrophils measured during a memory response (open symbols) were 1.3 to 2.4 higher than those of a primary response (closed symbols) (Figure 2B left; 2C, upper and middle panels and S2). In addition, the MFI of ROS^+^ ly6C^+^ monocytes was 1.2 to 1.5 fold increased compared to that of the ROS^+^ neutrophils and macrophages –at least the population of macrophages that we could recover from the spleen, suggesting that ly6C^+^ monocytes were intrinsically able to produce higher level of ROS than other effector phagocytes (Figure 2B, right panels). Such augmented effector activities depended on memory CD8^+^ T cells and CCL3 since immune mice treated either with an anti-CD8 depleting mAb or an anti-CCL3 neutralizing serum exhibited a lower frequency (∼30–40% instead of 60–70%) of ROS-producing inflammatory monocytes or neutrophils after challenge (white bars), equivalent to that found in primary infected animals (white and black bars) (Figure 2C, upper and middle panels).

Of note, while the subset of macrophages recovered from the spleen accounted for the large majority of ROS-producing phagocytes at steady state (data not shown), frequencies, numbers and MFI of these ROS^+^ macrophages did not vary neither during the primary nor the memory response (Figure 2B–C bottom panels and S2). Interestingly, whereas memory CD8^+^ T cells can promote ROS production by phagocytes, the frequencies of iNOS-expressing cells were similar in primary (closed symbols) and memory (open symbols) infected mice (Figure S3A). Moreover, in contrast to oxidative burst-deficient mice (Figure 1B & [6]), memory mice lacking the iNOS enzyme are equivalently protected during the secondary challenge as wt animals (white bars) and exhibited ∼5,000–6,500 fold less bacteria in the spleen than primary infected wt or iNOS^−/−^ mice (black bars) (Figure S3B). Therefore, memory CD8^+^ T cells allow (i) increased numbers of inflammatory monocytes and neutrophils to be activated and (ii) the differentiation of effector monocytes and neutrophils that produce higher quantities of ROS on a per cell basis.

**Figure 3:**
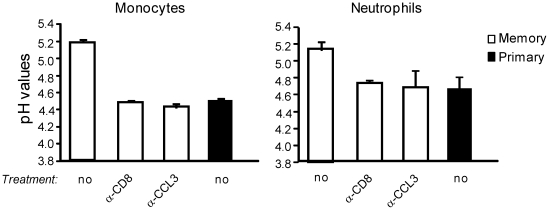
The phagosomal pH inside inflammatory monocytes and neutrophils rises during a secondary immune response. Primary (black bars) and memory mice (2–3/group) treated or not with anti-CD8 mAb or anti-CCL3 serum (white bars) were challenged with 3×10^5^ wt *Lm* for 6 hrs. Spleen cells were then incubated with latex beads coupled with pH sensitive and insensitive fluorochromes, stained for surface expression of Ly-6C and CD11b, and analyzed by FACS. Data show the pH values calculated for inflammatory monocytes and neutrophils in one (out of 2) independent experiments.

While an acidic luminal pH is essential for optimal activity of numerous microbicidal agents in macrophages and neutrophils, the NADPH-oxidase complex may also mediates the increase of the pH in phagocytic vacuoles that could lead under some experimental conditions to the activation of neutral proteases that kill microorganisms [14], [32]–[33]. Since phagocytes exhibited an increased production of ROS during a memory response, we monitored the phagosomal pH inside ly6C^+^ monocytes, neutrophils and macrophages from primary and memory mice 6 hrs after the infection (Figure 3, S4 and not shown). For this, latex beads were coated with pH-sensitive and pH-insensitive fluorescent dyes and incubated with splenocytes for 20 minutes to allow uptake of beads by phagocytes. Cells were further stained for the expression of different cell surface markers expressed by phagocytes and the fluorescence intensity of the phagocytosed beads was quantified by FACS. The ratio in fluorescence intensity between the two dyes directly reflected the pH inside the phagosomes as calibrated on a standard curve [34] (Figure S4A). We found that inflammatory monocytes which produced the highest levels of ROS per cell (Figure 2B, left panel), exhibited increased pH (∼5.2) inside their phagosomes in memory as compared to those of primary infected mice (∼4.45) (Figure 3, left panel). As expected, the intra-phagosomal pH for neutrophils [32]–[33], was also augmented in memory (∼5.2) versus primary (∼4.6) infected animals (Figure 3, right panel) [32]–[33]. These results were in sharp contrast with the intra-phagosomal pH of the splenic macrophage subset, which remained acidic (Figure S4B).

Similar acidic pH values that reached pH ∼4.5 three hrs after phagocytosis have also been observed for the macrophage cell lines RAW264.7 and J774 *in vitro* under similar experimental conditions [35]. The higher pH of the phagosomes of inflammatory monocytes and neutrophils from infected memory mice depended on CCL3^+^ memory CD8^+^ T cells, since treatment of memory mice with an anti-CD8 depleting mAb or an anti-CCL3 neutralizing serum induced a pH drop equivalent to that of primary infected mice. Collectively, our results show that during a memory response, innate cellular effectors -inflammatory monocytes and neutrophils- exhibit an inhospitable environment inside their phagosomes that helps better clearance of bacteria and likely is more toxic for pathogens than in the course of a primary response.

### Efficient prevention of cytosolic escape of *Lm* in phagocytes from immunized mice

**Figure 4:**
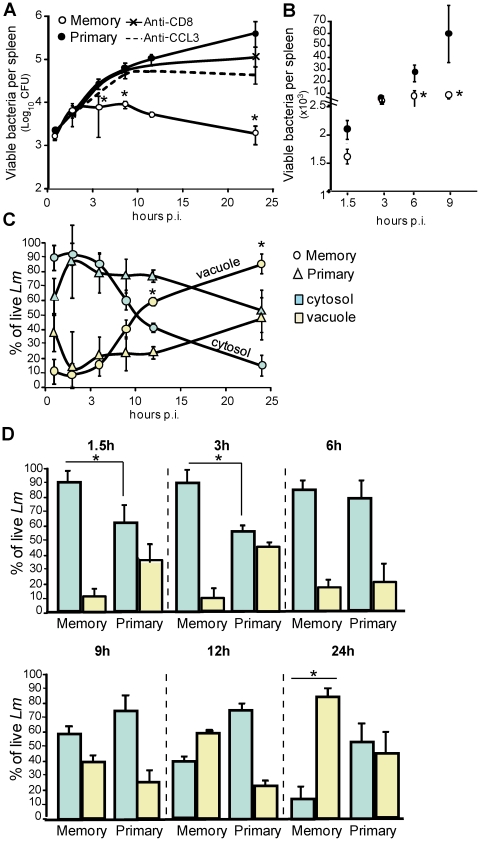
CCL3^+^ memory CD8^+^ T cells control bacterial growth during a secondary infection through a vacuolar and a cytosolic mechanism. (**A–B**) Primary and memory wt mice (10/group) were treated (**A**) or not (**B**) with anti-CD8 or anti-CCL3, and challenged with 3×10^5^ wt *Lm*. Mice were sacrificed at the indicated times after the infection. (**A–B**) show the number of bacteria CFUs (mean +/− SE) in the spleen in a pool of 2 replicate experiments. P values were calculated between groups of mice injected with PBS or immunized with wt *Lm* (n = 10). (**C–D**) Primary (triangles) and memory mice (rounds) (9–12/group) were challenged one month later with 3×10^5^ wt-L029 *Lm*. At the indicated times after infection, spleen cells treated or not with chloramphenicol, lysed and plated. (**C**) Graphs show the frequencies (mean +/− SE) of cytosolic (blue) and vacuolar (yellow) *Lm* CFUs in phagocytes in a pool of 3–4 experiments (n = 9). (**D**) is another representation of these data with histograms reporting frequencies (mean +/− SE) of cytosolic (blue bars) and vacuolar (yellow bars) *Lm* CFUs.

During *Lm* infection, the initial step of escape from the primary vacuole of phagocytosis to the cytosol of infected host cells is critical for bacterial survival [36]–[37]. Since *Lm* is rapidly trapped in such vacuoles [38], we reasoned that the most efficient way of controlling *Lm* growth is to prevent its escape to the cytosol of infected phagocytes. If this hypothesis is true, *Lm* multiplication between primary and memory infected animals would be expected to differ as early as during the first few hrs of infection. To investigate this possibility, we monitored *Lm* multiplication inside the spleen of primary and memory challenged animals that were concomitantly treated with an anti-CD8 depleting Ab, anti-CCL3 neutralizing serum or control goat serum (Figure 4 and not shown). As expected, primary or memory infected mice treated with anti-CD8 or anti-CCL3 did not control the infection. These mice exhibited 6.6 and 20 times more bacteria in the spleen respectively 9 and 12 hrs after the challenge infection than memory mice either untreated (white circles, plain line) or injected with control isotype matched Ab or serum (not shown). At 24 hrs, between 23 and 216 times more bacteria were measured in the groups of mice that did not control the infection (Figure 4A). Therefore, memory mice (open symbols) have already cleared 40%, 72% and 85% of the bacteria colony forming units (CFUs) 1.5, 6 and 9 hrs after the challenge infection as compared to primary infected animals (closed symbols) (Figure 4B). This result showed that *Lm* killing began very early after challenge of memory mice. Because several effector activities of phagocytes were improved at these time points, we hypothesized that killing of *Lm* could happen inside the primary vacuoles of phagocytes and with a higher efficacy in mice with memory responses.

**Figure 5:**
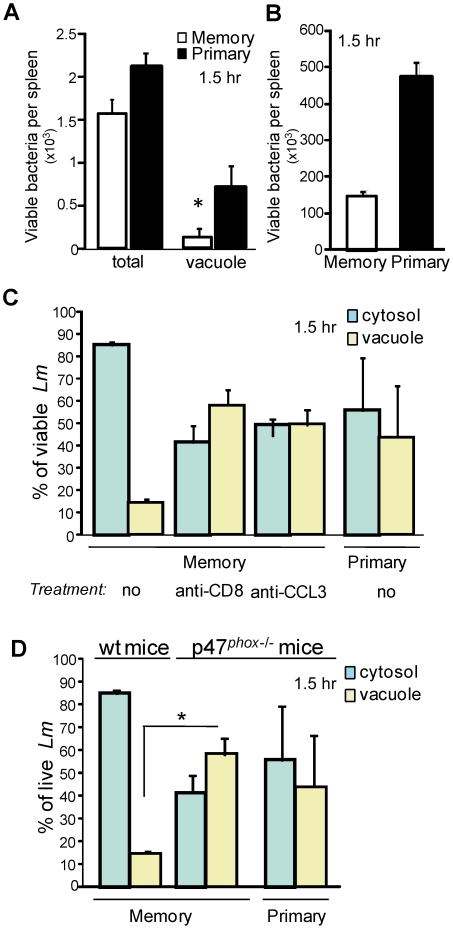
Early killing of *Lm* requires ROI and takes place inside the vacuoles of phagocytes from secondary infected mice. Primary and memory mice (9–12/group) were challenged one month later with 3×10^5^ wt-L029 *Lm* unless otherwise indicated. 1.5 hr post-infection, spleen cells treated or not with chloramphenicol, lysed and plated. (**A**) shows the number (mean +/− SE) of *Lm* CFUs recovered from the spleen. In (**B**), primary and immune mice (4–5/group) were challenged with 2×10^8^ ΔLLO *Lm*. The number of *Lm* CFUs (mean +/− SE) per spleen is shown in a pool of 2 experiments. In (**C**), primary and memory mice (4–6/group) were treated or not with anti-CD8 or anti-CCL3. Histograms show the frequencies (mean +/− SE) of cytosolic (blue bars) and vacuolar (yellow bars) *Lm* CFUs in phagocytes in a pool of 2 experiments. In (**D**), primary and memory p47*^phox^* ^−/−^ and wt C57BL/6 mice (5–6/group) were challenged with 3×10^5^ wt-L029 *Lm*. Histograms show the frequencies (mean +/− SE) of cytosolic (blue bars) and vacuolar (yellow bars) *Lm* CFUs in phagocytes in a pool of 2 experiments (n = 5).

To determine whether the early killing of Lm indeed takes place inside such vacuoles, we took advantage of a recombinant *Lm* (wt-L029) which expresses an antibiotic resistance to chloramphenicol upon reaching the cytoplasm of infected cells [39]. Splenocytes were incubated with chloramphenicol for a short period of time, which kills extracellular wt-L029 *Lm* and those that are inside vacuoles, only allowing survival of *Lm* that escaped to the cytosol (Figure S5). Primary and memory mice were challenged with wt-L029 *Lm* and the frequencies of vacuolar (chloramphenicol-sensitive) and cytosolic (chloramphenicol-resistant) viable bacteria inside phagocytes were determined 1.5, 3 and 6 hrs post-infection (Figure 4C–D). In primary infected mice, 60% of viable *Lm* localized to the cytosol and 40% to the vacuoles of cells 1.5 and 3 hrs later (Figure 4D). In contrast, while only 5–10% of the viable bacteria were found in the vacuoles of the phagocytes from memory mice (yellow bars), the vast majority (∼90–95%) were recovered from the cytosol (blue bars). At 6 hrs, bacterial localization was similar between primary and memory mice, with ∼90% of viable *Lm* localized to the cytosol and ∼10% to the vacuoles of phagocytes. Because the large majority of viable bacteria were recovered from the cytoplasm of infected cells in memory mice, these findings suggested that *Lm* was killed with a higher efficacy in the vacuoles of phagocytes from memory compared to primary infected mice. To provide further support to this hypothesis, we determined the absolute numbers of viable bacteria inside the vacuoles of phagocytes from primary and memory mice 1.5 hrs after the infection with wt-L029 *Lm* (Figure 5A). At this early time point following challenge, memory mice already exhibited ∼30–50% fewer bacteria in the spleen than primary infected mice. Moreover, memory mice exhibited two fold less viable bacteria in the vacuoles than animals with primary infections. To formally demonstrate the improved killing of Lm inside the vacuoles of phagocytes during the memory response, primary and memory mice were challenged with listeriolysin (LLO)-deficient (ΔLLO) *Lm* that remained trapped inside the vacuoles, and the number of viable *Lm* was determined 1.5 hrs later. As for wt *Lm*, memory mice infected with ΔLLO-*Lm* exhibited 2.5 times less viable bacteria than primary infected animals (Figure 5B).

To assess whether the improved killing of *Lm* occurring inside the vacuoles of the phagocytes resulted from the enhanced activity of the NADPH-oxidase complex during the memory response, primary or memory mice lacking the inducible p47^*phox*^ subunit of the NADPH-oxidase complex, or wt mice treated with either anti-CD8, anti-CCL3 or control Abs were infected with wt-L029 *Lm* (Figure 5C–D). p47*^phox^* ^−*/*−^ and wt memory mice treated with the anti-CD8 or the anti-CCL3 exhibited similar frequencies (∼40–60%) of viable vacuolar *Lm* as primary infected wt and p47*^phox^* ^−*/*−^ mice. Thus, taken together our results suggested that during a memory response, phagocytes are conditioned by the memory CD8^+^ T cells to generate a more effective bacteria-killing environment inside their early vacuoles of phagocytosis, and that this process is directly dependent upon increased activity of the NADPH-oxidase complex.

### Engulfment of cytosolic *Lm* in vacuolar structures of phagocytes from previously immunized mice

**Figure 6:**
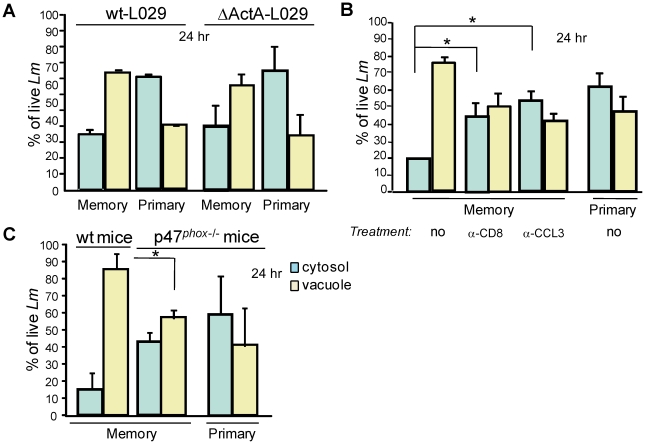
Cytosolic bacteria are engulfed and killed via ROI in the vacuoles of phagocytes during a protective secondary response. In (**A**), primary and immune mice (4–6/group) were challenged with 3×10^5^ wt- or ΔActA- L029 *Lm* and spleens were harvested 24 hrs later and treated as described in figure 5. Histograms show the frequencies (mean +/− SE) of cytosolic (blue bars) and vacuolar (yellow bars) *Lm* CFUs in phagocytes in a pool of 2 experiments. In (**B**), primary and memory mice (9–12/group) were treated or not with anti-CD8 or anti-CCL3 before challenge with 3×10^5^ wt-L029 *Lm*. Histograms indicate as in (A) in a pool of 3 experiments (n = 9). In (C), primary and memory p47*^phox^* ^−/−^ and C57BL/6 wt control mice (5–6/group) were challenged with 3×10^5^ wt-L029 *Lm*. Data indicate the number (mean +/− SE) of cytosolic and vacuolar *Lm* CFUs in phagocytes (n = 5).

While at very early time points after the infection (0–3 hrs), *Lm* was most efficiently killed within vacuoles of phagocytes from memory mice, the percentage of viable vacuolar bacteria (∼85%) and therefore the rate of phagosomal killing was comparable 6 hrs after the infection in primary and memory infected mice (Figure 4D). However, despite equivalent phagosomal elimination of *Lm*, the number of viable bacteria was significantly diminished in memory as compared to primary infected animals (Figure 4A–B). We therefore hypothesized that this either resulted from the more efficient early phagosomal killing of *Lm* or from other killing mechanisms distinct from those expressed inside the primary phagocytic vacuoles. If the first hypothesis were to be true, we expected to find a comparable distribution of the viable bacteria between the 2 groups of mice. We thus investigated the subcellular localization of *Lm* in infected splenocytes from primary and memory mice challenged with wt-L029 *Lm* (Figure 4C–D). Between 9 and 24 hrs after the infection, the relative proportion of viable cytosolic bacteria inside the phagocytes from memory mice drastically decreased from ∼60% to 15% compared to that of primary infected animals which only decreased about 20%. Viable Lm inside the cytosol of infected cells (i.e., chloramphenicol resistant) were rapidly lost and further recovered from vacuolar structures (i.e., chloramphenicol sensitive), suggesting that bacteria which had escaped to the cytosol of infected cells were rapidly re-engulfed into vacuoles. We reasoned that this could either result from *Lm* spreading from one cell to another or from the reinternalization of the free cytosolic Lm into phagosomal structures inside the same infected cell. To discriminate between the two possibilities, we used *Lm* lacking ActA that neither can spread from cell to cell nor infect neighboring cells. For that purpose, we generated an ActA-deficient L029 *Lm* strain that expressed the chloramphenicol resistance gene when reaching the cytosol of infected cells (Figure 6A). Upon inoculation of wt or ActA-deficient L029 *Lm*, phagocytes from primary and memory mice both exhibited similar frequencies of vacuolar viable bacteria, suggesting that the appearance of such vacuole-containing bacteria did not result from *Lm* spreading to neighboring cells but rather from Lm reinternalization inside the same infected cell.

To demonstrate that *Lm* relocalization from the cytosol to vacuolar structures indeed depended on CCL3^+^ memory CD8^+^ T cells, primary and memory mice were treated with an anti-CD8 depleting mAb, an anti-CCL3 neutralizing or control serum (not shown), and further challenged for 24 hrs with wt-L029 *Lm* (Figure 6B). CD8^+^ T cell depletion and CCL3 neutralization both prevented *Lm* engulfment into such vacuoles of phagocytes from memory mice that exhibited relative frequencies of cytosolic and vacuolar viable *Lm* similar to that of primary infected animals. Therefore, the subcellular localization of *Lm* was strongly modified during a memory response and this depended on memory CD8^+^ T cells and CCL3. This process also correlated with the more efficient bacterial killing occurring in memory mice compared to primary infected animals, which showed 1,920 and 415,200 bacterial CFUs respectively in the spleen 24 hrs after the infection (Figure 4). Overall, these data suggest that *Lm* destruction in the later phase of infection involves killing mechanisms distinct from those expressed in the primary vacuole of phagocytosis.

We next investigated how such *de novo* phagosomes that are likely key contributors to *Lm* destruction during recall infections were formed. Since ROS were required to clear *Lm* during a memory response (Figure 1B & [6]), we hypothesized that the production of ROS was also involved in *Lm* re-engulfment to these vacuolar structures of phagocytes from memory mice. For this, wt and p47*^phox-/-^* primary or memory mice were challenged with wt-L029 *Lm* and 24 hrs later the distribution of viable vacuolar and cytosolic bacteria was analyzed (Figure 6C). p47 *^phox-/-^* memory mice exhibited similar frequencies of viable Lm in the vacuoles and the cytosol of phagocytes compared to primary infected p47 *^phox-/-^* and wt control animals. Therefore, our results show that phagocytes from memory mice receive signals that license them to become more efficient to clear *Lm* by distinct mechanisms, amongst which is the engulfment of *Lm* into *de novo* formed phagosomal structures. This mechanism requires the generation of an oxidative burst promoted *via* the release of CCL3 by memory CD8^+^ T cells. Of note, we did not find any substantial differences in the frequencies of phagocytes undergoing cell death (Annexin V^+^, propidium iodide^+^) between primary and memory infected mice (Figure S6), likely ruling out increased phagocyte death in memory mice as a mechanism for controlling bacterial growth.

### Activation of autophagy in inflammatory monocytes and neutrophils by memory CD8^+^ T cells

**Figure 7:**
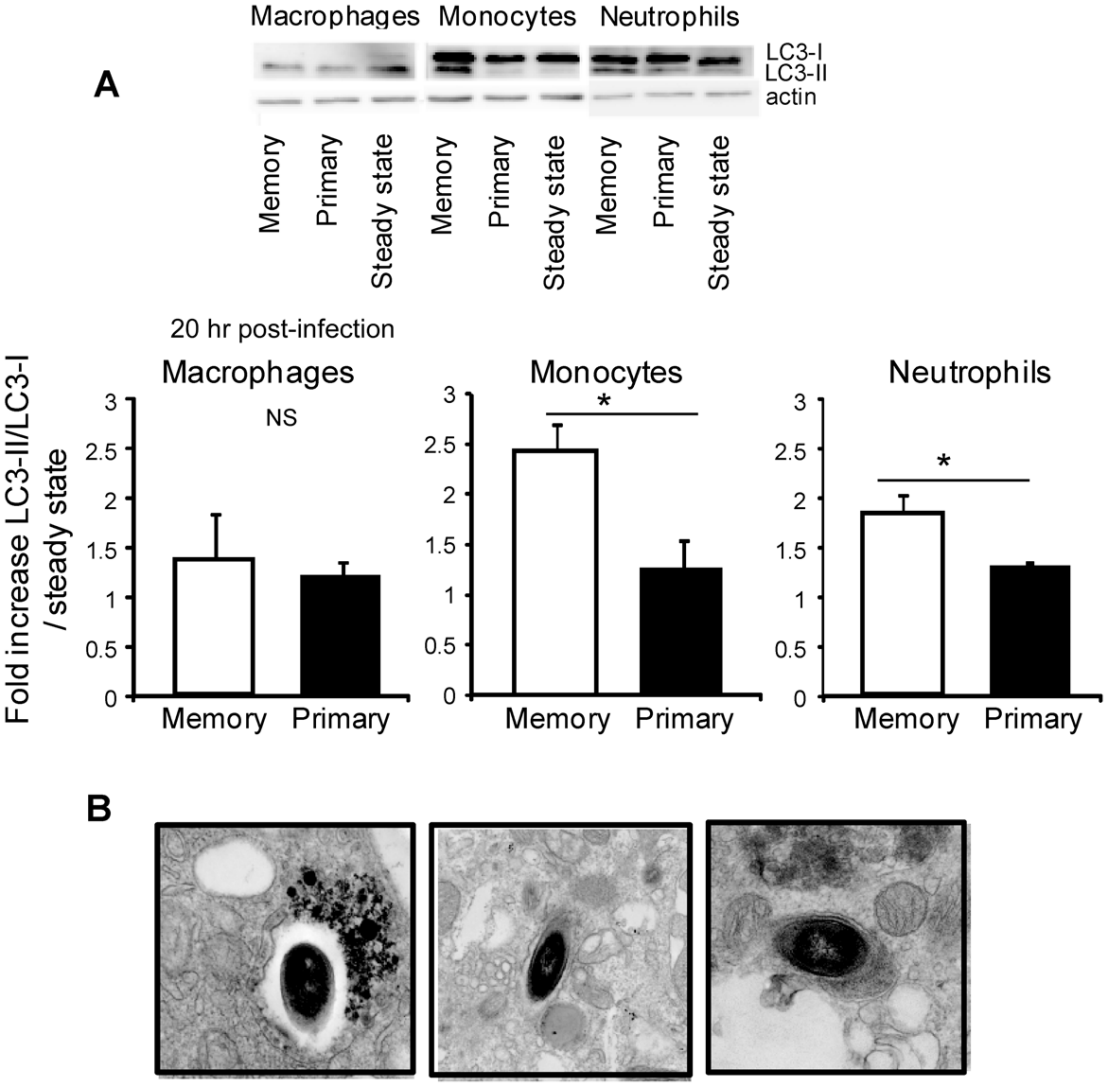
The reactive oxygen species generated inside inflammatory monocytes and neutrophils from memory mice induce autophagy. (**A**) Primary (black bars) and memory mice (white bars) (25/group) were challenged or not with 3×10^5^ wt-L029 *Lm*. 20 hrs after the infection, spleen cells (from 5 mice/group) were pooled, and flow-sorted inflammatory monocytes, neutrophils and macrophages lysed and each lysate separated on 15% SDS–PAGE and subsequently analyzed with anti-LC3 and anti-actin mAbs (control). (**A**) Data show representative images (upper) and the quantification histograms (lower) depicting the fold increase of LC3-II/LC3-I ratios (mean +/− SE) measured for each condition and cell subsets compared to the steady state in a pool of 2 to 5 independent experiments. In (**B**) primary and memory mice (7/group) were challenged with 5×10^5^ wt *Lm* expressing GFP. 20 hrs later, spleen cells (5 mice/group) were pooled and flow-sorted CD11b^+^GFP^+^ infected cells were fixed with glutaraldehyde, and analyzed by electron microscopy. Representative EM images of autophagosomes formation exhibiting double membranes around the bacteria are shown.

*Lm* has been shown to be a target for autophagy, a process that can be promoted by the generation of ROS [20], [40]. Since we observed that *Lm* is engulfed in vacuolar structures of infected phagocytes in a CCL3/ROS-dependent manner, we hypothesized that the phagocytes from memory mice were also primed to kill *Lm* by autophagy. We therefore monitored the conversion of the well-characterized autophagy marker LC3 inside the phagocytes. During this cellular process, cytosolic-free LC3-I is converted to lipidated LC3-I (LC3-II) that is covalently linked to phospholipids and associated with autophagosomes. This modification alters LC3-I gel migration properties which can be visualized by electrophoresis and western blotting. We assessed the ratio of LC3-II and LC3-I inside inflammatory monocytes, neutrophils and population of macrophages that extracted from the spleens of naïve, primary and memory mice infected for 20 hrs (Figure 7A). In contrast to these macrophages which demonstrated intrinsically high levels of autophagy in naïve uninfected mice (bottom blot), LC3-II/LC3-I ratios in inflammatory monocytes and neutrophils purified from the spleen of memory mice (white bars) were 2 and 1.3 times increased as compared to those from primary infected mice (black bars). This increase in LC3-II/LC3-I ratios most likely reflected the direct consequence of LC3-I lipidation and site specific proteolysis, e.g., conversion of LC3-I into LC3-II that localizes at the membranes of autophagosomes. Of note, we did not observe any significant increase in levels of LC3-I (Figure S7), suggesting that its rate of production was not affected by infection. Using electron microscopy, we looked for the presence of autophagosomes in phagocytes purified by cell sorting from memory mice infected with *Lm* expressing GFP for 20 hrs [22]. Figure 7B shows representative pictures of infected phagocytes containing *Lm* engulfed inside vacuolar structures that exhibit the characteristic double membrane feature of autophagosomes.

To formally link the induction of autophagy in monocytes and neutrophils from memory mice with the generation of an oxidative burst and the presence of CCL3^+^ memory CD8^+^ T cells, wt control or p47*^phox^* ^−*/*−^ primary and memory mice were treated with an anti-CD8 mAb or an anti-CCL3 serum and challenged with wt *Lm* (Figure S8 and not shown). CD8^+^ T cell depletion, neutralization of CCL3 or p47*^phox^* deficiency all prevented the induction of autophagy in inflammatory monocytes and neutrophils as compared to relevant controls. Collectively, our results show that innate inflammatory monocytes and neutrophils were able, with the help of the CCL3^+^ memory CD8^+^ T cells, to specifically induce efficient autophagosome formation during a memory response, and this allows the rapid control of the growth and the clearance of intracellular *Lm*.

## Discussion

Our study highlights that several effector functions of phagocytes are strongly modulated by memory CD8^+^ T cells in the course of a recall infection *in vivo* and that this leads to most efficient pathogen destruction. We show that bacterial killing involves activation of higher numbers of monocytes and neutrophils, which produce higher levels of ROS on a per cell basis during the secondary compared to the primary infection. ROS are likely inducing the rapid increase of the pH of the primary vacuoles of phagocytosis, as well as augmented levels of cellular autophagy, both allowing for *Lm* clearance. Collectively our results show that the cells from the innate immune system behave differently in the course of a recall infection by integrating signals from memory CD8^+^ T cells which stimulate them to express optimized antimicrobial effector functions. Recent studies also documented enhanced effector/memory CD4^+^ T cell-mediated clearance of pathogens by innate immune cells [8]–[9]; it is therefore conceivable that comparable antimicrobial protective mechanisms inside effector phagocytes are involved. Along similar lines, an elegant study documented that during lymphochoriomeningitis-induced meningitis, effector CD8^+^ T cells can promote the recruitment of pathogenic monocytes and neutrophils, leading to accelerated fatal outcome [41].

The current dogma is that only classical T and B lymphocytes that belong to the adaptive immune system are able to differentiate into long-lived memory cells exhibiting qualitatively improved functional properties. In fact, immunological memory is reflected by a stronger proliferation and expression of several effector functions, such as the secretion of specific cytokines and chemokines and the cytolysis of infected cells. In contrast, effector cells of the innate immune system like monocytes, macrophages and neutrophils are believed to mediate a rapid and antigen non-specific immune response that is qualitatively and quantitatively comparable each time the same pathogen is encountered. Macrophages have nevertheless been shown to undergo an adaptive-like response by acquiring distinct patterns of TLR-induced chromatin modifications that include modifications associated with the priming of antimicrobial effectors [42]. In the present work, we found that production of a chemokine by memory CD8^+^ T cells induced a strikingly distinct response of inflammatory monocytes and neutrophils. Indeed, we observed higher frequencies of ROS-producing inflammatory monocytes and neutrophils, an improved capacity to produce ROS and the upregulation of autophagy, a process also involved in intracellular pathogen clearance. Therefore, our data and that from others [7], [9], [41] promote the concept that the innate immune response during a secondary antigenic encounter can be regulated, either in a cell autonomous manner [42] or in response to lymphocyte-derived cues.

Interestingly, the licensing phenomenon we describe here seems exclusively observed for inflammatory monocytes and neutrophils and likely not for the population of macrophages extracted from the spleen. One explanation could be that the primary function of monocytes and neutrophils is to circulate via the blood and the lymphatic system and patrol the body to permanently sense the tissue environment in search for danger signals [29], [43]. Together, these cell types represent the vast majority of the blood-derived leucocytes and are rapidly attracted to damaged or infected tissues. Another possibility is that splenic monocytes, that may represent an important reservoir of resident monocytes possibly distinct from the blood monocytes, have intrinsically different functional properties [44]. Monocytes indeed exhibit a very dynamic plasticity and are able to differentiate into several functionally distinct subsets of effector cells within inflamed tissues [45]. Therefore, it is conceivable that these cells are more sensitive to cytokine/chemokine-mediated modulation of their fate in vivo compared to tissue-resident macrophages, which are terminally differentiated cells usually involved in steady-state tissue homeostasis.

What mechanisms are responsible for the improved immune response of innate phagocytes during secondary *Lm* infection? We recently found that during the recall infection, memory CD8^+^ T cells form transient “effector clusters” with inflammatory monocytes and neutrophils in the spleen [46]. These observations provide a spatio-temporal explanation to the subsequent cascade of effector functions that ultimately result in an efficient control of *Lm* burden during recall infection. In such clusters, memory CD8^+^ T cell-derived cytokines are concentrated, which likely favors the rapid and efficient integration of inflammatory signals by the phagocytes.

At the molecular level, it will be interesting to investigate whether the augmented production of ROS observed in inflammatory monocytes and neutrophils results from increased phosphorylation of the cytosolic regulatory protein p47*^phox^* leading to NADPH oxidase activation [47]. The improved response of phagocytes could then be attributed to post-translational regulation mechanisms. ROS, and more specifically O_2_^−^, can act as signaling molecules to induce cellular autophagy [40], a mechanism that is increasingly described as an important antimicrobial effector mechanism to kill pathogens. The increased level of ROS produced in the course of a memory response can also induce higher pH inside the vacuoles of inflammatory monocytes and neutrophils, and better bacterial killing. Higher pH can induce ion fluxes across the vacuolar membrane, which displaces strongly bound enzymes from the negatively charged proteoglycan granule matrix and allows them to digest and kill the microorganisms [14].

Collectively our data illustrate that the innate immune response and several antimicrobial effector functions of phagocyte populations can be regulated in the course of a memory response. ROS generation induced upon CCL3 secretion by reactivated memory CD8^+^ T cells likely provides the major signaling molecules promoting the expression of antimicrobial activities of both inflammatory monocytes and neutrophils. As we previously found, such processes also mediate the bystander killing of an unrelated intracellular pathogen [6]. Since we and others have shown that the modulation of the functions expressed by these phagocytes is potentially important for immunotherapeutic and vaccination strategies, it is critical to achieve a better knowledge on how such innate effector cells function in the context of distinct infections.

## Materials and Methods

### Ethics statement

This study was carried out in strict accordance with the recommendations in the Guide for the Care and Use of Laboratory Animals of the Committee of Animal Care and Use of the Regional Cote d'Azur. The protocol was approved by the Committee on the Ethics of Animal Experiments of the Institut de Pharmacologie Moléculaire et Cellulaire where the study was performed (Permit Number: B-06-152-5, delivered by the Veterinary Services of the Alpes-Maritimes Prefecture) and by the animal use committees at the Albert Einstein College of Medicine. All efforts were made to minimize suffering and provide humane treatment to the animals included in the study.

### Mice

Wt BALB/cByJ (Charles River Labs) and C57BL/6J (Jackson labs) 6–8 wk-old female mice, were used in all experiments unless otherwise indicated. p47*^phox^* ^−/−^ mice were obtained from European Mouse Mutant Archive (EMMA) and CX3CR1^+/−^ mice [48] on the C57BL/6 background were obtained from F. Geissmann's laboratory (King's College London, London). iNOS^−/−^ mice were purchased from CDTA (CNRS, Orléans). Transgenic mice were housed and bred in our SPF animal facility.

### Bacteria

The *Listeria monocytogenes (Lm)* 10403 s wt strain was used in all experiments. The wt-GFP, ΔLLO, wt-L029 and ΔActA *Lm* are on the 10403 s background and have been previously described [22], [39]. The ΔActA-L029 was generated by transfection of the pLCR plasmid that expresses the chloramphenicol resistance gene under the ActA promoter. The wt-L029 *Lm* and the pLCR plasmid are a kind gift from Dan Portnoy (UCSB, CA, USA). The wt strains exhibit a LD50 of 3×10^4^ in BALB/c mice. HKLM was prepared as previously described [49]. All Lm were prepared from clones grown from organs of infected mice and kept at −80°C.

### Infection of mice and measure of protective immunity and mice survival

For *Lm* infections, bacteria were grown to a logarithmic phase (OD_600_ = 0.05–0.15) in Broth Heart Infusion medium (Sigma), diluted in PBS and injected i.v. into lateral tail vein. In all experiments, mice were primary immunized with a 0.1xLD_50_ of bacteria (3×10^3^). Secondary infections were carried out one month later with indicated bacteria. To measure *Lm* titers in spleen and liver, organs were dissociated in 0.1% X-100 Triton (Sigma) and serial dilutions plated onto BHI media plates. To determine the number and frequency of viable *Lm* localized inside the vacuole or the cytosol, spleens were dissociated in RPMI1640 (Gibco) 5% FCS and each cell suspension was split in two and either incubated or not with 10 µg/ml of chloramphenicol at 37°C for 45 min. After centrifugation, cell pellets were resuspended in 0.1% triton X-100 and serial dilutions plated onto BHI media plates. The next day, we determined the total number (vacuolar and cytosolic) of viable bacteria by counting the colony forming units (CFU) from untreated samples. The number of cytosolic bacteria was determined by counting the CFU from chloramphenicol-treated cells. By subtracting these numbers, we obtained the number of vacuolar bacteria. *Lm* CFUs in these cells were determined as described above.

### *In vivo* neutralization and depletion experiments

For neutralization of CCL3, mice were treated with 75 µg of anti-CCL3 or goat IgG control i.v at the time of the secondary infection and 24 h later. For neutralization of IFN-γ, mice received one injection of 500 µg of the mAb XMG1.2 concomitantly to the recall infection. For depletion of CD8^+^ T cells, mice were injected daily 3 times with 100 µg of the anti-CD8β H-35 mAb or its control isotype i.p and further infected one day after.

### Antibodies and reagents

Anti-F4/80 (A3-1)-fluorescein isothiocyanate (FITC) was purchased from Caltag Laboratories. The following mAbs were purchased from BD Pharmingen:, anti-CD11b (M1/70) -phycoerythrin (PE), -peridinin chlorophyll protein (PcP) or -allophycocyanin (APC), anti-TCR-PE (H57-597), anti-NK1.1-PE (PK136), anti-CD19-PE (MB19-1), anti-Ly-6G-PE (1A8), anti-Ly-6C-FITC and biotine (AL-21), anti-CD11c-PE and APC (HL3), anti-TNF-α-APC (MP6-XT22) and control rat IgG_1_ mAb, AnnexinV and propidium iodide. Anti-NOS-2 (M-19) polyclonal rabbit Abs were purchased from Santa Cruz Biotechnology. Goat anti-rabbit-Alexa647 was from Molecular Probes.

### Cell suspensions

Organs were cut in small pieces and incubated at 37°C for 20 min in HBSS medium (Gibco) containing 4000 U/ml of collagenase I (Gibco) and 0.1 mg/ml of DNase I (Roche). Red blood cells were lysed for 2–3 min in 170 mM NH_4_Cl, 17 mM Tris HCl pH 7.4.

### Antibody staining and flow cytometry

Cells were stained with the specified Abs in PBS 0.5% of BSA (FACS buffer). For intracellular TNF-α staining, splenocytes were incubated at 37°C 5%CO_2_ for 3–4 hrs in RPMI1640 (Gibco) 5% FCS, 2 µg/ml Golgi Plug (BD Pharmingen) with or without 5×10^8^ HKLM/ml. Cells were incubated for 20 min on ice with the indicated cell surface marker mAbs, fixed in 1% PFA FACS buffer for 20 min on ice, permeabilized for 30 min in 1XPerm/Wash (BD Pharmingen). For intracellular staining of TNF-α cells were incubated for 20 min on ice in FACS buffer containing anti-TNF-α or control rat IgG_1_. For intracellular staining of iNOS, cells were incubated for 20 min on ice in FACS buffer containing anti-iNOS rabbit polyclonal, or control normal goat IgG and staining was revealed using goat anti-rabbit Alexa647 mAb. In all cases, cells were washed, fixed for 30 minutes in 1% PFA FACS buffer and analyzed on a FACScalibur cytofluorometer (Becton Dickinson, BD). When indicated, cells were sorted on a FACSvantage SE cell sorter (Becton Dickinson).

### Assay for the production of ROS

5−10×10^6^ splenocytes were incubated for 3 h at 37°C and 5%CO2 in RPMI1640 containing 5% FCS with 5×10^8^ HKLM/ml and 160 µM hydroethidine (Polysciences). Hydroethidine is oxidized by ROS in red fluorescent ethidium bromide (EB) therefore allowing for the detection of ROS-producing cells. Cells were washed in FACS buffer and stained for expression of cell surface markers.

### Measure of phagosomal pH

This protocol was adapted from Savina et al. [34]–[35]. 50 to 70 µl of 3 micrometers of polybeads amino (Molecular probes) were covalently coupled with 50 µl of 50 mg/ml FITC (pH sensitive) (Fluorescein isothio isomer 1, Sigma F7250) and 50 µl of 1 mg/ml FluoProbes 647 (pH insensitive) (Fluo Probes, Molecular Probes FP-AK7740) in 400 µl of sodium hydrogen carbonate buffer at pH 8.0 for 2 h at room temperature. After extensively washing with glycine 100 mM, the beads were suspended in PBS. Cells were then pulsed with the coupled beads for 20 min, extensively washed in cold PBS and stained on ice for CD11b, Ly-6C or F4/40 surface markers and immediately analyzed by FACS, by selectively gating on the cells that have phagocytosed one latex bead. The ratio of the mean fluorescence intensity (MFI) emission between the two dyes was determined. Values were compared with a standard curve obtained by resuspending the cells that had phagocytosed beads for 20 min at a fixed pH (ranging from pH 5.5 to 8.0) and containing 1XPerm/Wash (BD Pharmingen). The cells were immediately analyzed by FACS to determine the emission ratio of the two fluorescent probes for each pH value.

### Western blot analysis

PBS-injected or wt immunized BALB/c, C57BL/6 wt or p47*^phox^* ^−/−^ mice were sacrificed and spleen cells were positively enriched using anti-CD11b-specific MACS beads (Myltenil) according to the standard manufacturer protocol and further flow-cell sorted on expression of CD11b, F4/80 and Ly-6G cell surface markers. Inflammatory monocytes were defined as CD11b^med/high^Ly-6G^-^F4/80^med^ cells, neutrophils as CD11b^high^Ly-6G^high^F4/80^-^ cells and macrophages as CD11b^med^Ly-6G^med^F4/80^high^ cells (purity>87%). The same amount of flow-sorted cells (2−4×10^6^) were washed in PBS and pellets were resuspended in lysis buffer pH 8.0 (Tris-HCl 50 mM, NaCl (200 mM), EDTA 5 mM, 0.5% Triton X-100, 0.5% deoxycholic acid) for 45 min at 4°C. Homogenates were centrifuged for 15 min at 20,000xg and lysate supernatants were analyzed by SDS-PAGE, transferred to PVDF membranes, blocked and probed with a rabbit anti-LC3 (2 µg/ml) (Novus Biologicals), a rabbit anti-actin (0.5 µg/ml) (Sigma), a anti-rabbit peroxidase (1∶10,000) (Beckman Coulter). Western blot quantification was performed with the ImageJ software.

### Electron microscopy

Flow-sorted CD11b^+^GFP^+^ infected cells were fixed with 1.6% glutaraldehyde in 100 mM phosphate buffer pH 7.5. Cells were incubated at 4°C in 100 mM cacodylate buffer pH 7.0 containing 1% osmium tetroxyde. Cellular pellets were washed with distilled water and incubated for 2 hrs with 0.5% uranyl acetate buffer at room temperature in the dark. Cells were washed in water; pellets were dehydrated in increasing acetone series and embedded in epoxy resins. Blocks were thin-sectioned using standard procedures and contrasted for the observation on a Philips CM12 electron microscope.

### Statistics

Statistical significance was calculated using an unpaired Mann-Whitney test and Instat software, except if specified differently in the legend of the figure. All P values of 0.05 or less were considered significant and are referred to as such in the text.

## Supporting Information

Figure S1 **Analysis of iNOS expression and TNF-α secretion by inflammatory Ly-6C^+^ monocytes during primary and secondary** ***Lm*** **infection.** Primary and memory C57BL/6 wt and CX_3_CR1^eGFP+/−^ mice were infected with 3×10^5^ wt *Lm* for 10 h (**A, B**) or 24 h (**B**). Spleen cells were restimulated with HKLM and analyzed by FACS for CD11c, CD11b, Ly-6C, CX3CR1eGFP surface expression and intracellular TNF-α and iNOS. Data show in (**A**) are representative FACS profiles of TNF-α and iNOS production by CD11c^low^CD11b^+^ Gr1^+^ /ly6C^+^ TCR^neg^, CD19^neg^, NK1.1^neg^, Ly-6G^neg^ spleen cells (highlighted in red) and are representative of 2 independent experiments with n = 6–10 mice/group. In (**B**), bar graph shows the frequency of splenic monocytes producing TNF-α among total monocytes in a pool of 3 independent experiments with n = 9–12 mice/group. P values between the different conditions are indicated with *P<0.05.
(TIF)

Figure S2 **Increased numbers of ROS^+^ inflammatory monocytes and neutrophils during secondary infection.** Mice (9–10 per group) injected with PBS (closed symbols, primary) or 0.1xLD_50_ (3×10^3^) wt *Lm* (white symbols, memory) were challenged 30 days later with 10xLD_50_ (3×10^5^) wt *Lm*. At the indicated times after challenge, spleen cells were restimulated with Heat Killed *Lm* (HKLM) in the presence of hydroethidine (HE) and analyzed by FACS for CD11b and Ly-6C expression. Data show the number of phagocytes in spleen (mean +/− SE) (upper panel) and the number (mean +/− SE) (bottom panel) of ROS-producing phagocytes and are representative of a pool of 2–3 replicate experiments. P values between the different conditions are indicated with *P<0.05, **<0.01, ***<0.001.
(TIF)

Figure S3 **iNOS expression by phagocytes is similar during primary and secondary** ***Lm*** **infection and is not required for protection.** (**A**) Primary (closed circles) and memory mice (3–5/group) (open circles) were challenged with 3×10^5^ wt *Lm*. At indicated times after infection, spleen cells were analyzed by FACS for cell-surface CD11b and Ly-6C and intracellular iNOS. Data show the frequencies (mean +/− SE) of iNOS-producing cells per spleen and are representative of 2 independent experiments. (**B**) Primary (black bars) and memory mice (white bars) (3–5/group) were challenged with 3×10^5^ wt *Lm* for 2 days. Data show the number of bacteria CFUs (mean ± SE) in the spleen in a representative (out of two) experiment. P values between the different conditions are indicated.
(TIF)

Figure S4 **The pH values measured inside the phagosomes from macrophages of memory and primary infected mice are similar.** (**A**) Spleen cells from mice were incubated with latex beads coupled with a pH sensitive (FITC) and insensitive (APC) fluorochrome and stained for surface expression of Ly-6C and CD11b. Cells were then resuspended in medium of fixed pH (ranging from pH 4.5 to 8.0) and with 0.1% Triton X-100 and immediately analyzed by FACS. Data show standard curve of the emission ratio of the two dyes obtained for a given pH. (**B**) Primary (black bars) and memory mice (2–3/group) treated or not with anti-CD8 or anti-CCL3 (white bars) were challenged with 3×10^5^ wt *Lm* for 6 h. Spleen cells were then incubated with latex beads coupled to the two dyes as above and stained for surface expression of Ly-6C and CD11b. Data show the pH values calculated for macrophages in one (out of 2) independent experiment.
(TIF)

Figure S5 **Schematic representation of experimental design allowing for discrimination between vacuolar and cytosolic bacteria.**
(TIF)

Figure S6 **Phagocytes do not undergo substantially different cell death in primary versus memory challenged mice.** Primary and memory mice (4/group) were challenged 4 wks later with 3×10^5^ wt *Lm*. 24 hrs post-infection, death of phagocytes, e.g., monocytes and neutrophils (as defined earlier) was measured using annexin V and propidium iodide costaining, according to the manufacturer protocol. Data are representative of 1 out of 2 replicate experiments with similar results.
(TIF)

Figure S7 **The increased LC3-II/LC3-I ratios in inflammatory monocytes and neutrophils results from LC3-I conversion in LC3-II during a secondary infection.** Primary (black bars) and memory mice (white bars) (25/group) were challenged or not with 3×10^5^ wt-L029 *Lm*. 20 h after the infection, spleen cells (5 mice/group) were pooled, flow-sorted inflammatory monocytes, neutrophils and macrophages lysed and lysates separated on 15% SDS–PAGE and subsequently analyzed with anti-LC3 and anti-actin (control) mAbs. Data show histograms (mean +/− SE) representing the fold increase of LC3-I and LC3-II obtained for each condition and cell subsets compared to steady state in a pool of 2 to 5 experiments (n = 5). P values between the different conditions are indicated.
(TIF)

Figure S8 **The induction of autophagy in inflammatory monocytes and neutrophils depends on CCL3^+^ memory CD8^+^ T cells-induced ROS.** Primary and memory mice, wt and p47*^phox^* ^−/−^ C57BL/6 mice (10–15/group) treated or not with anti-CD8 or anti-CCL3 were challenged or not with 3×10^5^ wt-L029 *Lm.* 20 hrs after infection, spleen cells (5/group) were pooled, and flow-sorted inflammatory monocytes, neutrophils and macrophages lysed and lysates separated on 15% SDS–PAGE and subsequently analyzed with anti-LC3 and anti-actin (control) mAbs. In (**A**), histograms (mean +/− SE) represent the fold increase of LC3-II/LC3-I ratios for monocytes purified from anti-CD8- (upper panel), anti-CCL3-treated mice (bottom panel) and control-treated versus uninfected mice. In (**B**) histograms (mean ± SE) represent the fold increase of LC3-II/LC3-I ratios for each cell subsets purified from wt and p47 phox^−/−^ versus uninfected mice in a pool of 2–3 experiments.
(TIF)
