# Supplementary figures and images for: Inflammatory Monocytes and Neutrophils Are Licensed to Kill during Memory Responses In Vivo
Source: PLoS Pathog. 2011 Dec 29;7(12):e1002457. doi: 10.1371/journal.ppat.1002457 (PMC3248567; doi:10.1371/journal.ppat.1002457)

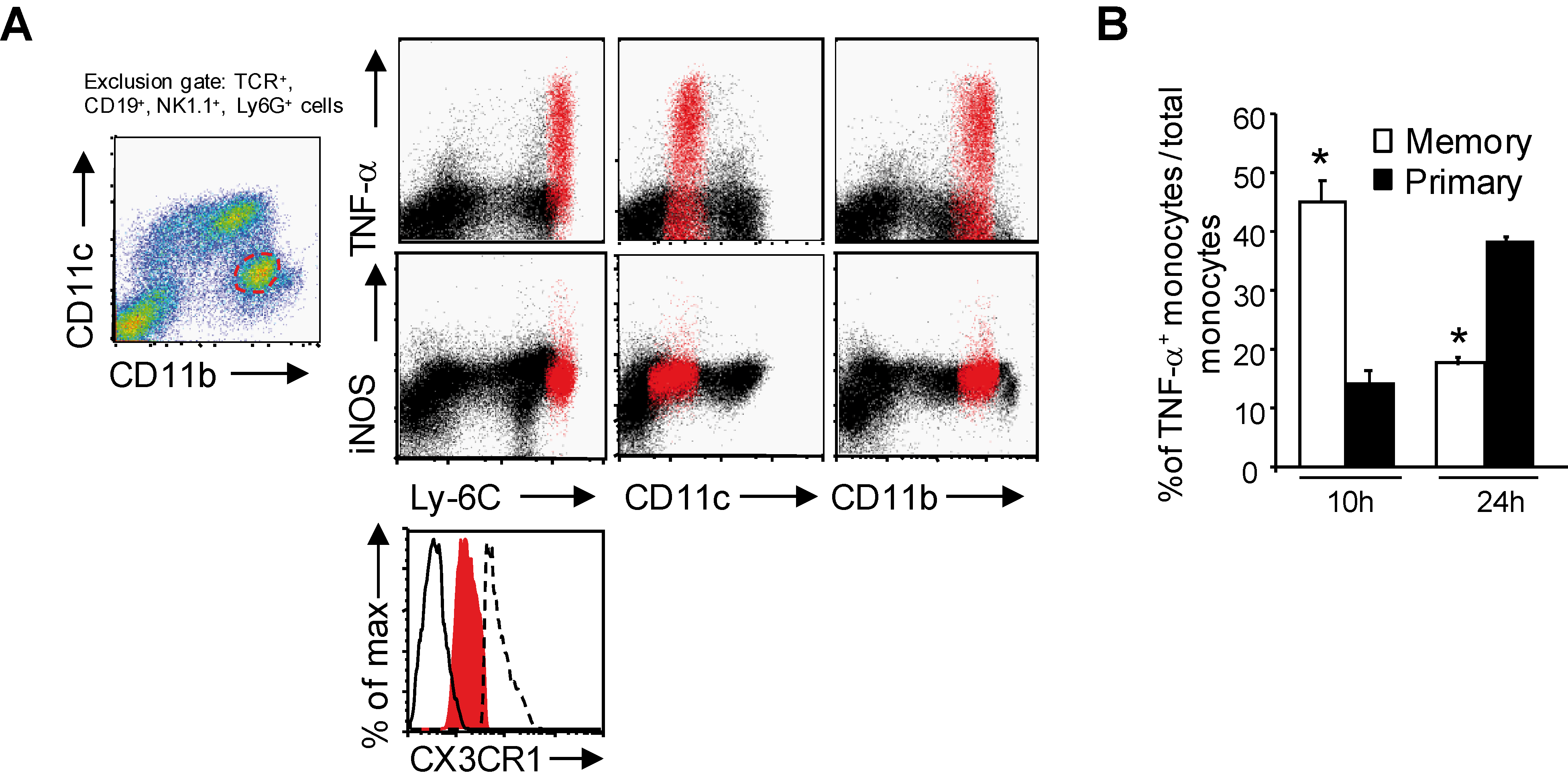

Supplement: Figure S1 — Analysis of iNOS expression and TNF-α secretion by inflammatory Ly-6C+ monocytes during primary and secondary Lm infection. Primary and memory C57BL/6 wt and CX3CR1eGFP+/− mice were infected with 3×10^5^ wt Lm for 10 h (A, B) or 24 h (B). Spleen cells were restimulated with HKLM and analyzed by FACS for CD11c, CD11b, Ly-6C, CX3CR1eGFP surface expression and intracellular TNF-α and iNOS. Data show in (A) are representative FACS profiles of TNF-α and iNOS production by CD11clowCD11b+ Gr1+ /ly6C+ TCRneg, CD19neg, NK1.1neg, Ly-6Gneg spleen cells (highlighted in red) and are representative of 2 independent experiments with n = 6–10 mice/group. In (B), bar graph shows the frequency of splenic monocytes producing TNF-α among total monocytes in a pool of 3 independent experiments with n = 9–12 mice/group. P values between the different conditions are indicated with *P<0.05. (TIF) [file ppat.1002457.s001.tif]

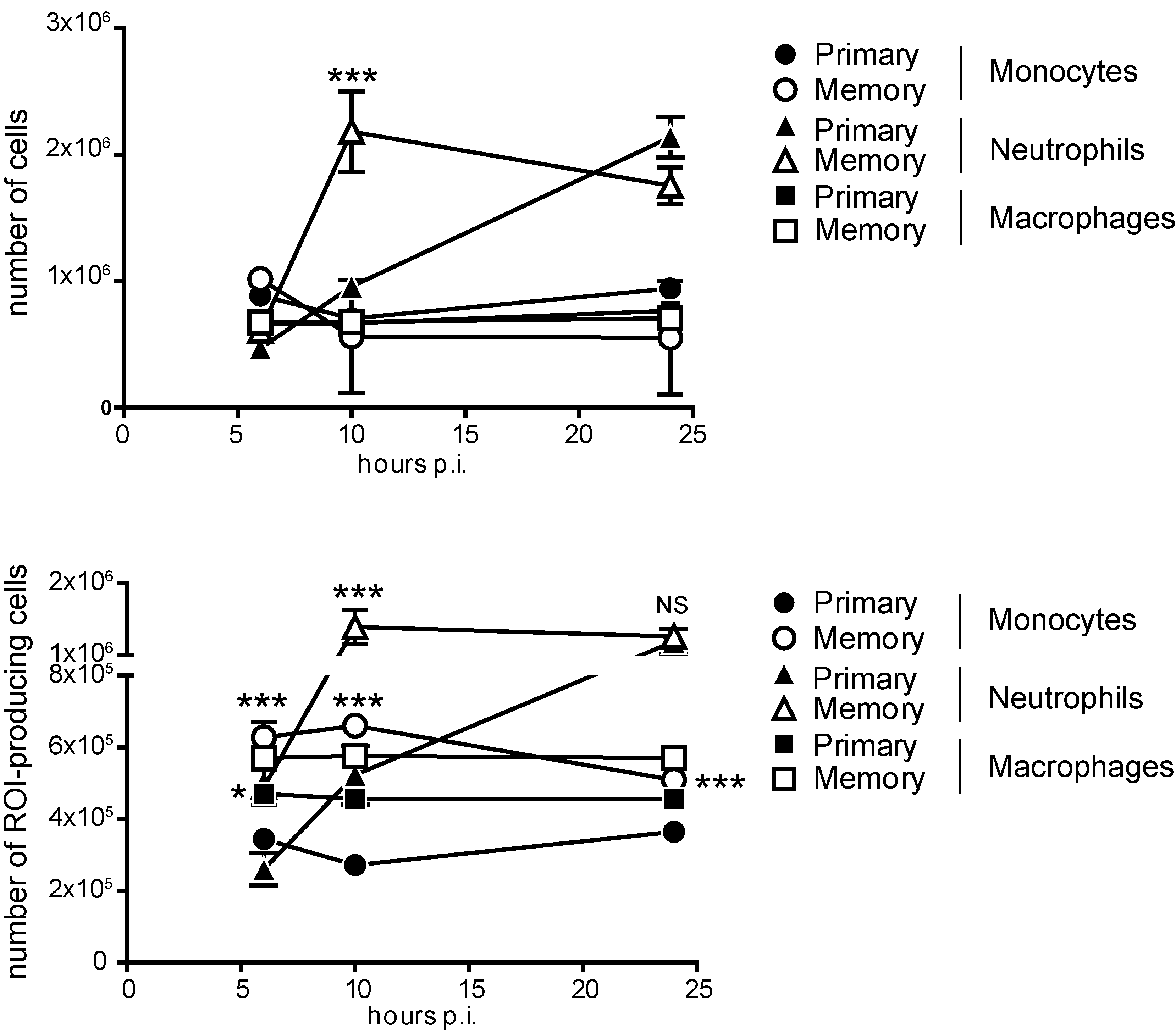

Supplement: Figure S2 — Increased numbers of ROS+ inflammatory monocytes and neutrophils during secondary infection. Mice (9–10 per group) injected with PBS (closed symbols, primary) or 0.1xLD50 (3×10^3^) wt Lm (white symbols, memory) were challenged 30 days later with 10xLD50 (3×10^5^) wt Lm. At the indicated times after challenge, spleen cells were restimulated with Heat Killed Lm (HKLM) in the presence of hydroethidine (HE) and analyzed by FACS for CD11b and Ly-6C expression. Data show the number of phagocytes in spleen (mean +/− SE) (upper panel) and the number (mean +/− SE) (bottom panel) of ROS-producing phagocytes and are representative of a pool of 2–3 replicate experiments. P values between the different conditions are indicated with *P<0.05, **<0.01, ***<0.001. (TIF) [file ppat.1002457.s002.tif]

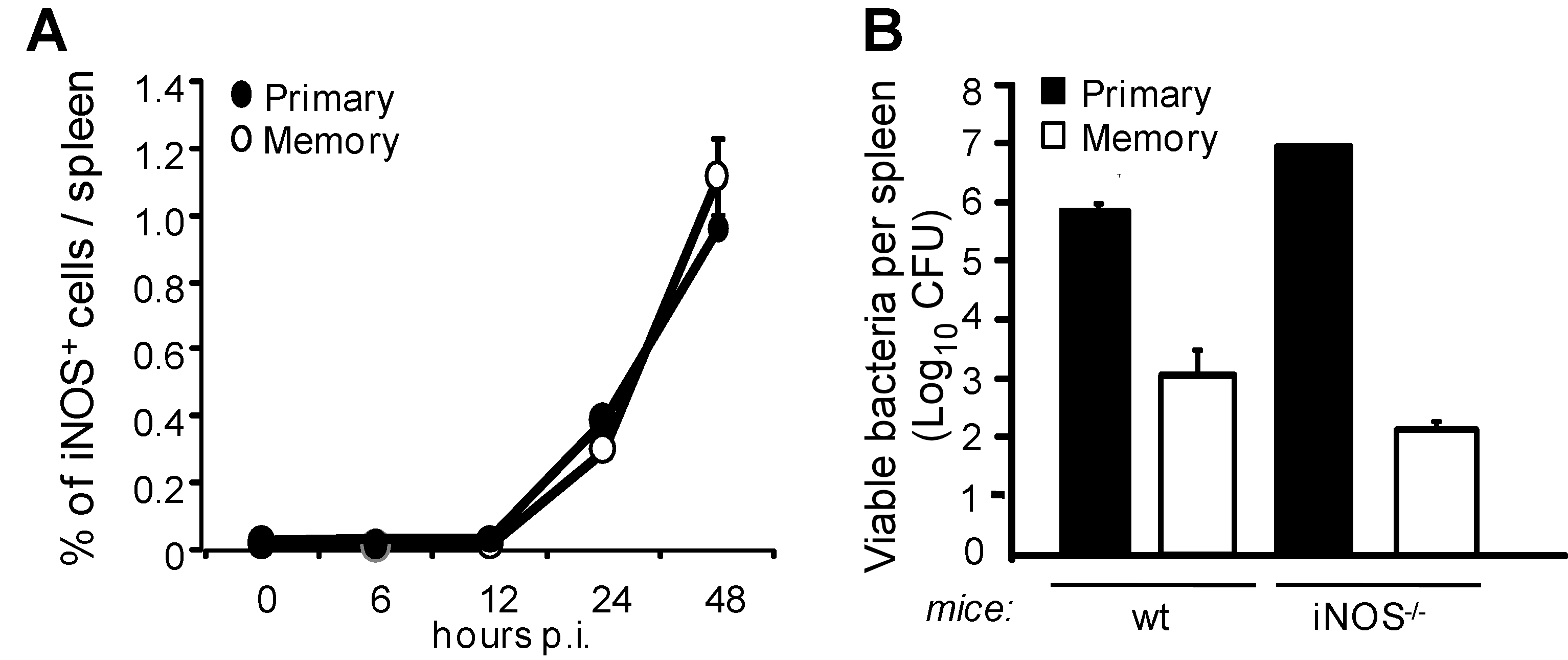

Supplement: Figure S3 — iNOS expression by phagocytes is similar during primary and secondary Lm infection and is not required for protection. (A) Primary (closed circles) and memory mice (3–5/group) (open circles) were challenged with 3×10^5^ wt Lm. At indicated times after infection, spleen cells were analyzed by FACS for cell-surface CD11b and Ly-6C and intracellular iNOS. Data show the frequencies (mean +/− SE) of iNOS-producing cells per spleen and are representative of 2 independent experiments. (B) Primary (black bars) and memory mice (white bars) (3–5/group) were challenged with 3×10^5^ wt Lm for 2 days. Data show the number of bacteria CFUs (mean ± SE) in the spleen in a representative (out of two) experiment. P values between the different conditions are indicated. (TIF) [file ppat.1002457.s003.tif]

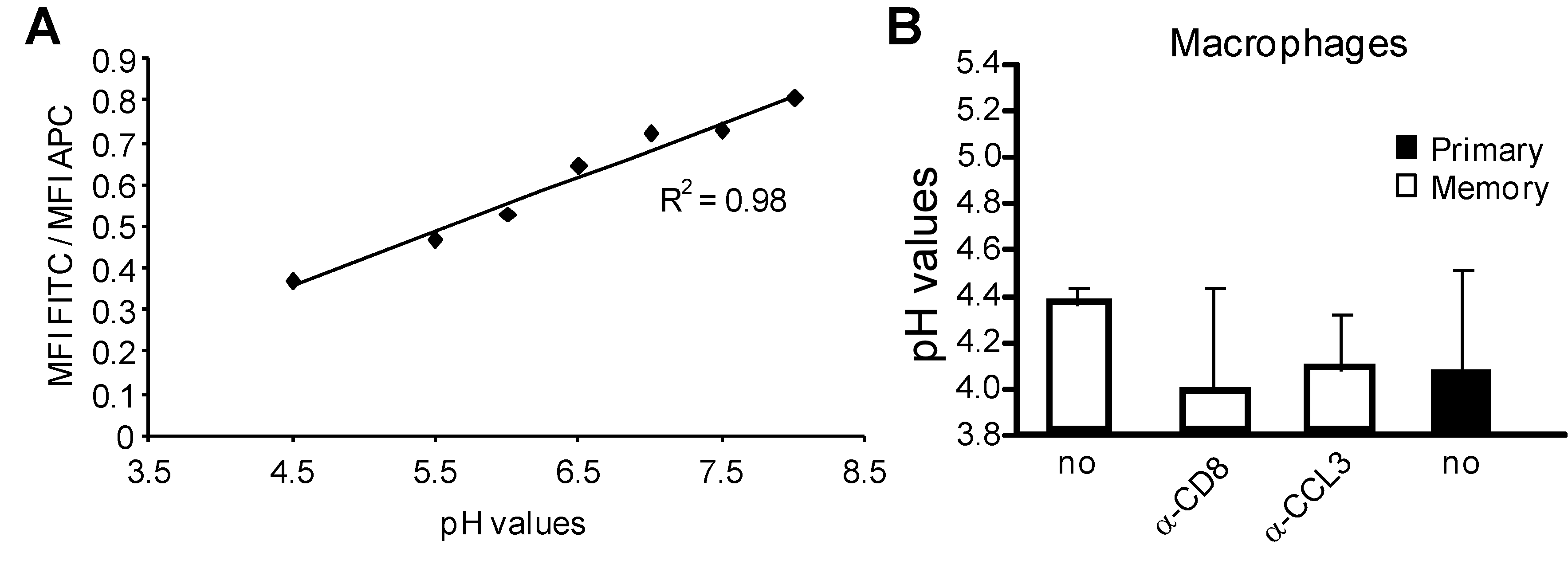

Supplement: Figure S4 — The pH values measured inside the phagosomes from macrophages of memory and primary infected mice are similar. (A) Spleen cells from mice were incubated with latex beads coupled with a pH sensitive (FITC) and insensitive (APC) fluorochrome and stained for surface expression of Ly-6C and CD11b. Cells were then resuspended in medium of fixed pH (ranging from pH 4.5 to 8.0) and with 0.1% Triton X-100 and immediately analyzed by FACS. Data show standard curve of the emission ratio of the two dyes obtained for a given pH. (B) Primary (black bars) and memory mice (2–3/group) treated or not with anti-CD8 or anti-CCL3 (white bars) were challenged with 3×10^5^ wt Lm for 6 h. Spleen cells were then incubated with latex beads coupled to the two dyes as above and stained for surface expression of Ly-6C and CD11b. Data show the pH values calculated for macrophages in one (out of 2) independent experiment. (TIF) [file ppat.1002457.s004.tif]

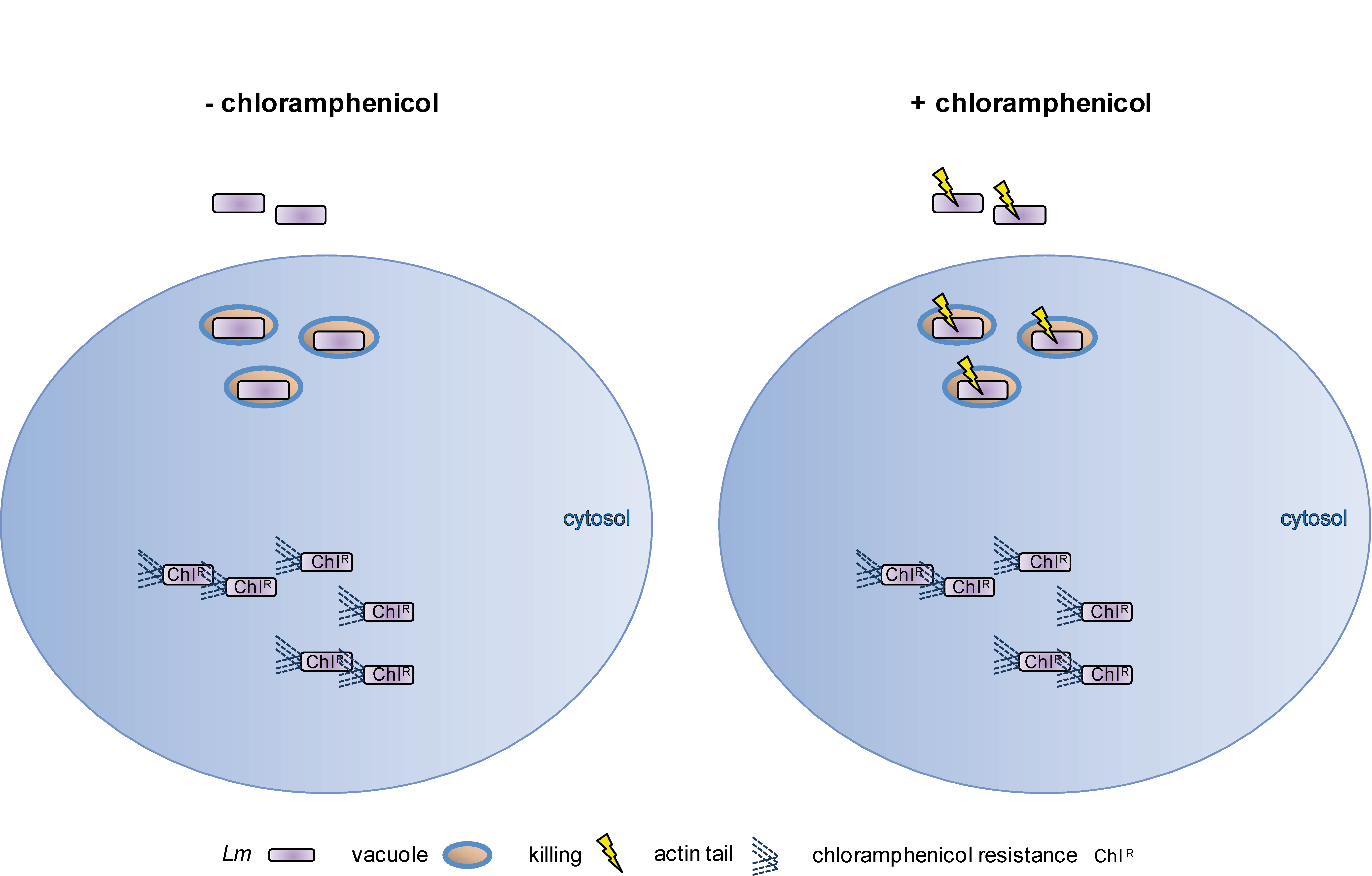

Supplement: Figure S5 — Schematic representation of experimental design allowing for discrimination between vacuolar and cytosolic bacteria. (TIF) [file ppat.1002457.s005.tif]

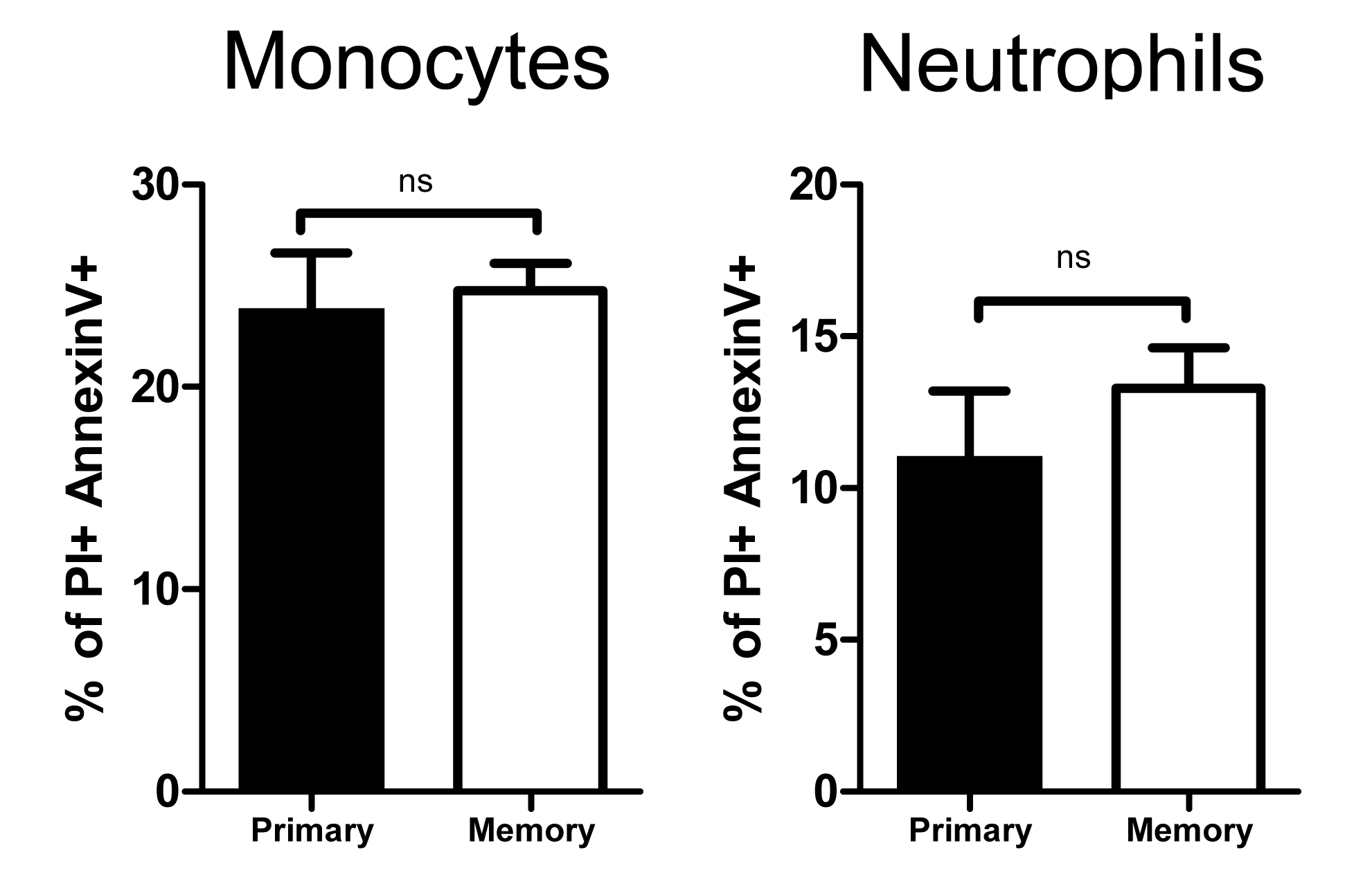

Supplement: Figure S6 — Phagocytes do not undergo substantially different cell death in primary versus memory challenged mice. Primary and memory mice (4/group) were challenged 4 wks later with 3×10^5^ wt Lm. 24 hrs post-infection, death of phagocytes, e.g., monocytes and neutrophils (as defined earlier) was measured using annexin V and propidium iodide costaining, according to the manufacturer protocol. Data are representative of 1 out of 2 replicate experiments with similar results. (TIF) [file ppat.1002457.s006.tif]

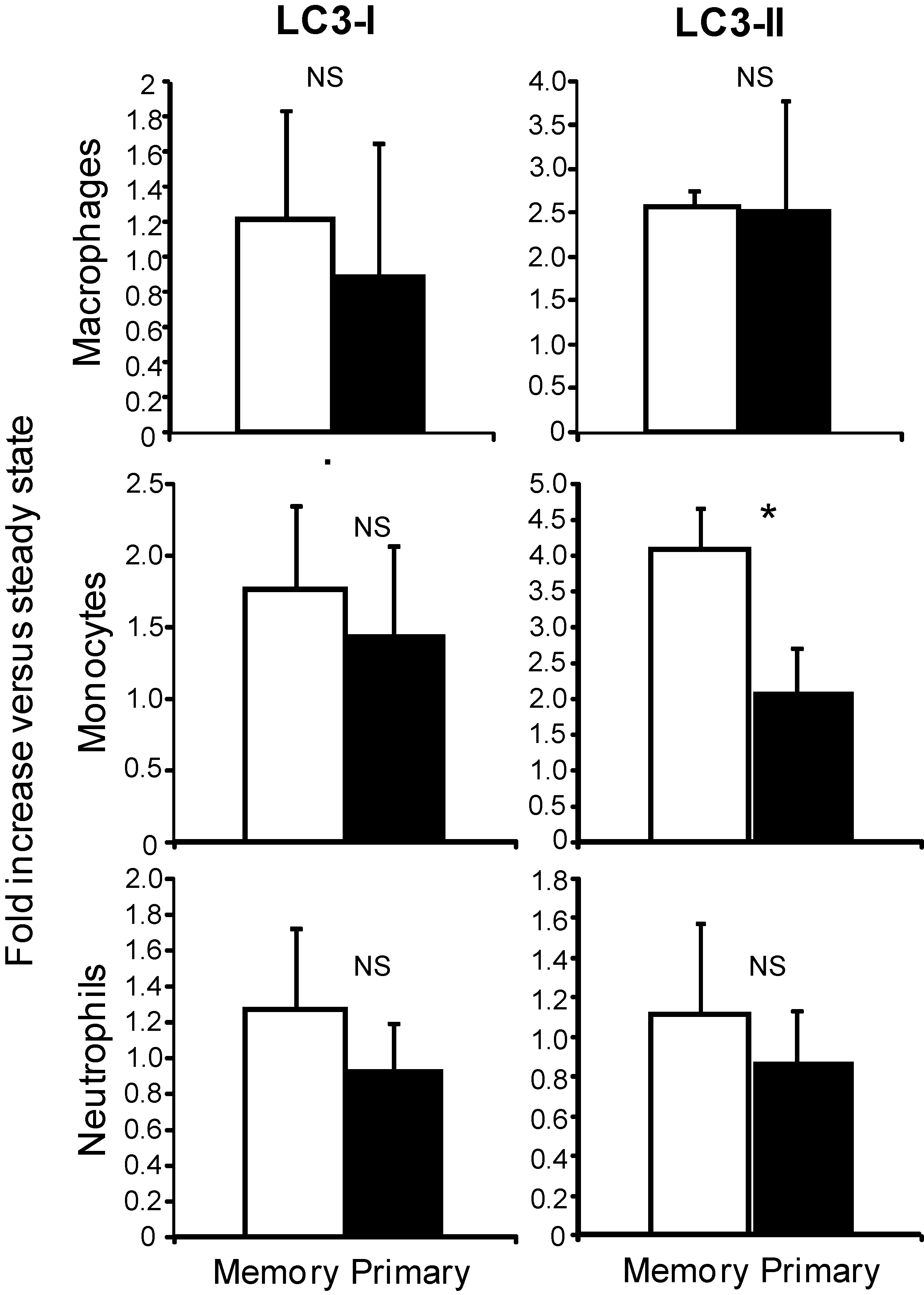

Supplement: Figure S7 — The increased LC3-II/LC3-I ratios in inflammatory monocytes and neutrophils results from LC3-I conversion in LC3-II during a secondary infection. Primary (black bars) and memory mice (white bars) (25/group) were challenged or not with 3×10^5^ wt-L029 Lm. 20 h after the infection, spleen cells (5 mice/group) were pooled, flow-sorted inflammatory monocytes, neutrophils and macrophages lysed and lysates separated on 15% SDS–PAGE and subsequently analyzed with anti-LC3 and anti-actin (control) mAbs. Data show histograms (mean +/− SE) representing the fold increase of LC3-I and LC3-II obtained for each condition and cell subsets compared to steady state in a pool of 2 to 5 experiments (n = 5). P values between the different conditions are indicated. (TIF) [file ppat.1002457.s007.tif]

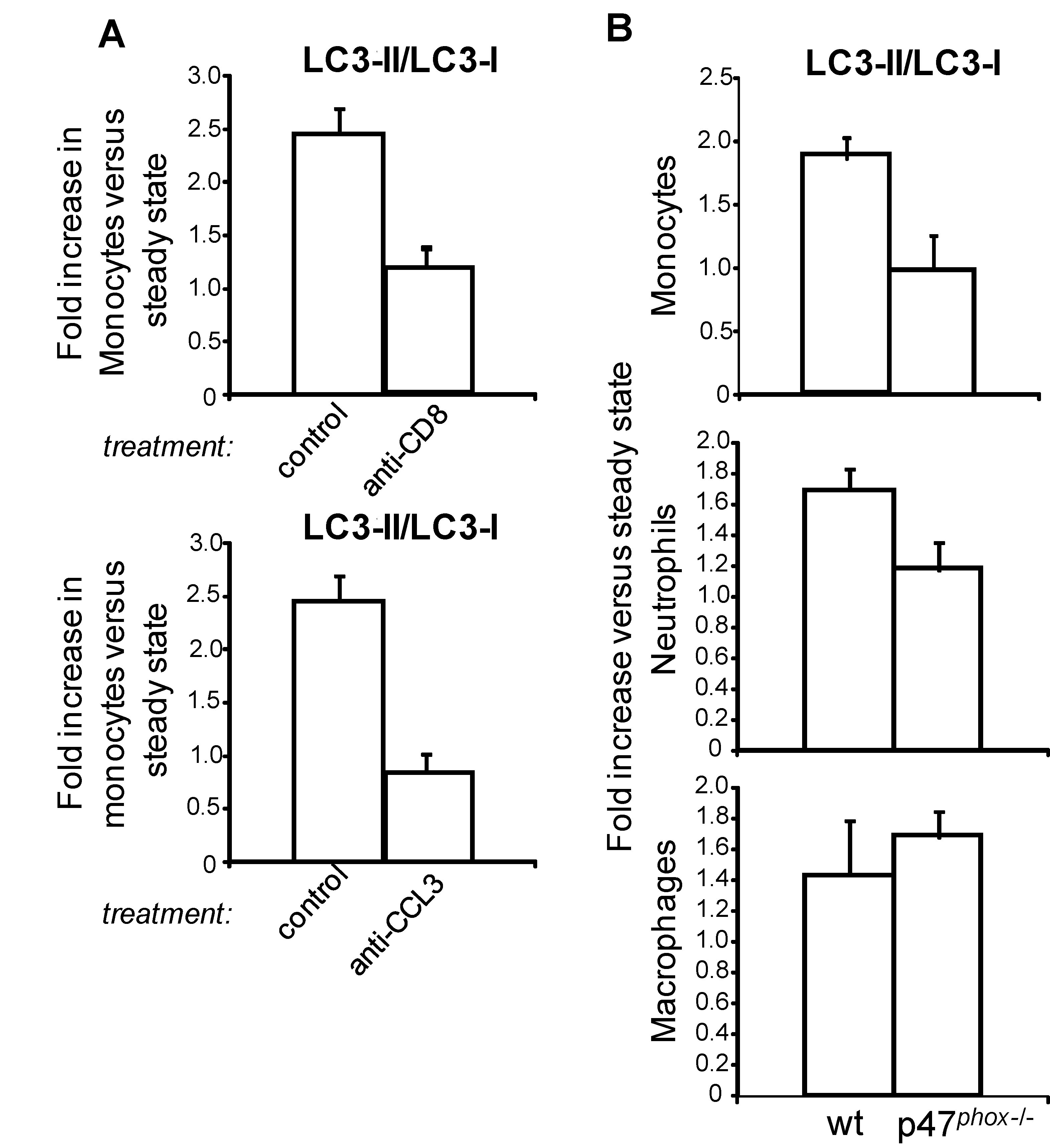

Supplement: Figure S8 — The induction of autophagy in inflammatory monocytes and neutrophils depends on CCL3+ memory CD8+ T cells-induced ROS. Primary and memory mice, wt and p47phox −/− C57BL/6 mice (10–15/group) treated or not with anti-CD8 or anti-CCL3 were challenged or not with 3×10^5^ wt-L029 Lm. 20 hrs after infection, spleen cells (5/group) were pooled, and flow-sorted inflammatory monocytes, neutrophils and macrophages lysed and lysates separated on 15% SDS–PAGE and subsequently analyzed with anti-LC3 and anti-actin (control) mAbs. In (A), histograms (mean +/− SE) represent the fold increase of LC3-II/LC3-I ratios for monocytes purified from anti-CD8- (upper panel), anti-CCL3-treated mice (bottom panel) and control-treated versus uninfected mice. In (B) histograms (mean ± SE) represent the fold increase of LC3-II/LC3-I ratios for each cell subsets purified from wt and p47 phox−/− versus uninfected mice in a pool of 2–3 experiments. (TIF) [file ppat.1002457.s008.tif]
